# Associations between organizational readiness and implementation fidelity in national implementation of WHO’s labour care guide in Sweden: a prospective observational study

**DOI:** 10.1186/s43058-026-01073-z

**Published:** 2026-08-14

**Authors:** Johanna Sjöberg, Anna Ramö Isgren, Thomas Abrahamsson, Marie Blomberg, Kristin Thomas

**Affiliations:** 1 Department of Biomedical and Clinical Sciences, Linköping University, Linköping, Sweden; 2 Clinical Department of Obstetrics and Gynecology in Linköping, Region Östergötland, Linköping, Sweden; 3 Clinical Department of Obstetrics and Gynecology in Norrköping, Region Östergötland, Norrköping, Sweden; 4 Crown Princess Victoria Children’s Hospital, Linköping, Sweden; 5 Department of Medicine, Health and Caring, Linköping University, Linköping, Sweden; 6 Department of Medicine, Karolinska Institutet, Huddinge, Sweden

**Keywords:** Implementation, Organizational readiness, Fidelity, LCG, LCG-SE

## Abstract

**Background:**

During 2024 and 2025, an adapted version of the World Health Organization’s Labour Care Guide was implemented in approximately half of Sweden’s labor wards within the PICRINO trial. Organizational readiness is widely regarded as a key determinant of successful implementation, however, empirical evidence linking organizational readiness to implementation outcomes remain limited. This study aimed to examine the association between organizational readiness for implementing the Labour Care Guide in Sweden and implementation fidelity, to explore variations in readiness and fidelity across organizational and individual factors and to assess changes in fidelity over time.

**Methods:**

The study included the implementation of the Labour Care Guide at ten labor wards in Sweden. Organizational readiness was measured among health care staff (*n* = 282) using the E-Ready 2.0 questionnaire before implementation was launched. Fidelity was assessed by analyzing degree of documentation within the Labour Care Guide tool at 3, 6, and 9 months (*n* = 750). A coding protocol including all seven sections of the tool, in which two sections were considered core components, was used to systematically analyze fidelity. Fidelity was then compared between wards with the aggregated highest, intermediate, and lowest levels of readiness at ward level.

**Results:**

The three wards reporting the aggregated highest levels of organizational readiness also demonstrated the highest implementation fidelity, while the three wards with the lowest reported aggregated readiness showed the lowest fidelity. In addition, staff at smaller labor wards reported greater readiness than staff at larger wards, and readiness was higher in non-university hospitals compared with university hospitals. Physicians reported higher readiness than midwives. Overall fidelity to the Labour Care Guide declined over time, however, this trend was not observed among core components.

**Conclusions:**

This study provides empirical support for an association between organizational readiness and implementation fidelity when introducing the Labour Care Guide in Sweden. Fidelity to core components remained stable, suggesting that essential elements were more resilient to implementation challenges. Findings highlight the importance of assessing and strengthening organizational readiness prior to implementation and of providing sustained support over time to maintain fidelity, particularly for non-core components.

**Trial registration:**

www.clinicaltrials.gov NCT05560802, 2022-09-17.

**Supplementary Information:**

The online version contains supplementary material available at 10.1186/s43058-026-01073-z.

Contributions to the literature • Although organizational readiness is widely theorized as a key determinant of implementation success, empirical evidence linking readiness to concrete implementation outcomes within clinical settings remains limited.

• This study, conducted in a high-income setting, across Swedish labor wards during the rollout of a clinical tool, provides evidence of an association between organizational readiness and implementation fidelity, strengthening the evidence for readiness as an actionable implementation determinant.

• By bridging theory and practice, this study advances implementation science. The findings underscore the importance of strengthening readiness when implementing innovations in healthcare, with implications for improving implementation quality, patient care, and public health outcomes.

## Background

During 2018, the World Health Organization (WHO) updated their recommendations concerning intrapartum care [1] and a new documentation tool for maternal health care staff was designed; the Labour Care Guide (LCG) [2]. Recently, the International Federation of Gynecology and Obstetrics (FIGO) recommended the LCG to be universally adopted and used in all settings [3]. To date, LCG implementation evaluations have mainly been conducted in low- and middle-income countries. Evidence on clinical outcomes remains inconclusive, with studies reporting both reduced and increased cesarean section rates, as well as varying effects on the rate of obstetric interventions [4–6]. Healthcare staff’s experiences of using LCG have been evaluated, showing generally high satisfaction with the tool [7]. However, the evaluation also concluded that improvements in usability are necessary. It is therefore recommended that implementation of the LCG tool is accompanied by training and supportive supervision to ensure readiness to implement the tool within clinical settings [7].

LCG has been translated and adopted to a Swedish digital version (LCG-SE), which has been implemented in just over half of Sweden’s labor wards through the trial *Can the use of a next generation Partograph based on WHO’s latest Intrapartum Care Recommendations Improve Neonatal Outcomes? A stepped-wedge cluster randomized trial* (PICRINO) [8]. If the trial demonstrates beneficial results, a nationwide rollout in Sweden could be recommended. Knowledge on implementation determinants and how uptake and fidelity to the documentation tool can be facilitated during such a large-scale, nation-wide implementation is needed, and could inform similar, large-scale implementation efforts internationally.

Organizational readiness for change, defined as the shared psychological and behavioral preparedness of organizational members (i.e., staff) to implement a specific change [9], is frequently considered in implementation theory as critical for successful implementation [10]. The empirical evidence regarding whether, or under which conditions, organizational readiness can be associated with implementation outcomes is however unclear, and further research is recommended. Known is, that contextual factors as well as characteristics of the innovation affect an organization’s readiness [11].

Fidelity, defined as the degree to which an innovation is implemented and used as prescribed by its developers, is one of the most commonly used outcome measures for evaluating implementation efforts [12]. Fidelity has been associated with intervention effectiveness where higher fidelity has shown to result in improved patient outcomes with decreased resource utilization and mortality in medical centers [13].

There is some evidence pointing to an association between organizational readiness and fidelity. For example, a study within healthcare specialty clinics aiming to evaluate the effectiveness of strategies intended to enhance readiness showed effects on early implementation outcomes [14]. Another evaluation, concluded that fidelity to guidelines for improving pain care at cancer centers could have been improved by considering the readiness for change of each clinic prior to implementation [15]. Also, it has been indicated within early childhood education and care [16], that clinics with higher levels of organizational readiness also had higher fidelity scores to a physical activity policy intervention. To the best of our knowledge, no studies on how organizational readiness influences implementation outcomes have been performed within maternity care. The PICRINO trial, with its carefully planned and rigorous implementation across multiple labor wards, provides a unique opportunity to examine organizational readiness and fidelity, their association, and variations across organizational and individual factors. The aim of this study was therefore to examine whether organizational readiness for implementing the digital LCG-SE tool within Swedish labor care was associated with implementation fidelity in terms of registrations within the documentation tool. The study also investigated variations in readiness and fidelity across organizational and individual factors and how the degree of fidelity developed over time.

## Theory and methods

### Design

This is a two-part prospective observational study combining a cross-sectional survey of organizational readiness as perceived by healthcare staff (*n* = 282) and a prospective observational assessment of the degree of fidelity to the digital LCG-SE documentation tool over time at 3 (*n* = 250), 6 (*n* = 250), and 9 months (*n* = 250). The study is reported in accordance with the STROBE guidelines for observational studies [17] and StaRI standards for reporting implementation studies [18].

### Organizational readiness for change

Organizational readiness for change refers to the extent to which members of an organization (e.g., healthcare staff) are psychologically and behaviorally prepared to implement a new practice (e.g., documentation tool) [9]. According to Weiner’s theory, readiness is a shared state characterized by collective commitment (willingness) to change and confidence in the organization’s capability to execute it. This construct is influenced by factors such as change valence (perceived value of the change), and situational factors like resources and task demands [9]. Scaccia and colleagues [19] further emphasize the role of organizational capacity, including leadership, culture, and infrastructure, as critical determinants of readiness. A core premise is that high readiness is associated with greater likelihood of successful implementation, as it shapes motivation and effort toward adopting and sustaining innovations [20].

### Implementation fidelity

Implementation fidelity refers to the degree to which an intervention (e.g., the LCG-SE documentation tool) is used as intended [21]. It is a critical construct in implementation science because high fidelity ensures that observed clinical outcomes can be attributed to the intervention rather than variations in its delivery or use. Fidelity can provide information about whether implementation strategies are sufficient, and if additional strategies are needed. Fidelity encompasses multiple components, including adherence, exposure, quality, and participant responsiveness. ‘Adherence’ refers to whether the implementation includes all important components, as planned by the original developers. ‘Exposure’ focuses on the quantity, frequency, duration, and sequence of the implemented intervention. ‘Quality’ reflects how intervention practices and core components are implemented, and ‘participant responsiveness’ demonstrates participants’ satisfaction and involvement [21].

Within the PICRINO trial, all participating labor wards received the same implementation support (e.g., on-site education for staff, induction day, super-users at each site) as described in detail in the study protocol [8]. Prior to the commencement of the trial, a designated group of healthcare staff translated and adapted the WHO user’s manual and the LCG into the digital LCG-SE, as well as the Swedish user’s manual, where all modifications from the original LCG are described. Accordingly, fidelity, in terms of adherence, was assessed as high. The same group also developed educational materials, provided on-site staff education, and maintained communication with the sites throughout the study period. Consistency within the group was ensured through regular meetings, and all sites were provided with the same implementation package. Thereby, fidelity, in terms of quality, was deemed comparable for all included sites. Regarding participants’ responsiveness, some fidelity framework developers consider readiness as being a part of that component [21]. This study therefore focused on assessing fidelity in terms of exposure.

### The labour care guide-Sweden documentation tool

The LCG-SE is a digital documentation tool designed to monitor and assess the active phase of labor [8]. To support clinical decision-making, the tool includes an alert feature that displays an attention symbol (a red circle) when a recorded measurement deviates from the normal range. This functionality aims to reduce unnecessary interventions while maintaining maternal and neonatal safety [2]. LCG-SE includes seven sections: (1) Background and labor characteristics, (2) Supportive Care, (3) Woman, (4) Baby, (5) Labor Progress, (6) Medication, and (7) Shared Decision-Making. Across these sections, the tool enables the documentation of 32 parameters.

For this study, fidelity was assessed as the degree of documentation in all sections of the LCG-SE tool over time. Fidelity to medical guidelines was beyond the scope of this analysis. Core components of the tool (defined as components with the greatest clinical relevance and unique to the intervention) [21] were defined prior to the study within the research group (JS, ARI, MB) as Section 2 (Supportive Care) and Section 7 (Shared Decision-Making). These sections were decided as core to the intervention, since they introduced parameters that were previously undocumented or unsystematically recorded in existing documentation systems. These sections, along with Section 5 (Labor Progress), were given extra attention during on-site education for staff. Section 5 was not considered a core component in this study, as its primary innovation was the incorporation of new medical guidelines rather than novel documentation features. Parameters in the other four sections (Section 1, 3, 4 and 6) were part of the assessment but decided non-core components since they largely reflected existing documentation practices.

### Research settings

Sweden is a high resource setting where labor care is free of charge, and most women give birth in hospitals. Midwives are the primary caregivers, assisted by nursing assistants. If complications arise, the obstetrician has medical responsibility, but teamwork with midwives and nursing assistants [22, 23]. The research settings for the implementation process were complex due to the wide-spread context including many sites, the new digital solution, and the fact that it affected the daily work for different professions. The study included the ten labor wards participating in the first two clusters of the PICRINO trial, implementing the LCG-SE during spring 2024. Collectively, these wards employ approximately 1300 health care professionals and manage around 18,000 births annually. The sites represent variation in organizational structure, clinic size, geographical location, and hospital type.

### Data collection on organizational readiness

Data on organizational readiness were investigated by the validated questionnaire E-Ready 2.0 [24], which was developed to measure organizational readiness to implement eHealth innovations within Swedish health care. The questionnaire assesses perceived readiness for change on both individual and collective levels specifically in terms of culture, capacity and motivation for change, leadership support, and perceived innovation characteristics. The questionnaire consists of 32 items and six subscales: (1) conditions for change at the workplace (Capacity), (2) individual conditions for change (Motivation), (3) support and engagement among management (Leadership), (4) readiness among colleagues (Culture), (5) consequences on status quo (Innovation characteristics), and (6) workplace attitudes (Motivation). In addition, three single statements investigate compatibility with current work routines (Innovation Characteristics), commitment to change (Motivation) and perceived need for change (Innovation Characteristics). The items measure agreement rated on 4-point (subscales 1–4, 6) or 5-point (subscale 5) Likert-scales. Items were modified by adding “*the tool Labour Care Guide Sweden (LCG-SE)*” or “*LCG-SE*” throughout. Also, items on gender, birth year, workplace, profession, employment rate, years worked at current workplace, and years in current profession were added. In total, the modified questionnaire included 39 items. The questionnaire was tested regarding face validity among midwives and nursing assistants, resulting in minor language modifications to the items. It was estimated to take approximately 10 min to complete.

The questionnaire was disseminated to all staff working with maternity care during the wash-out period of the PICRINO trial, defined as the period after on-site education, before official implementation launched (4–5 weeks). The questionnaire was accessible within the LCG-SE platform, enabling staff to complete the questionnaire when logged in to the system. A reminder was also sent to respondents’ work e-mail. Inclusion criteria were: staff working with labor care and having been employed for more than three months at a labor ward participating in the PICRINO trial.

A total readiness sum score (range from 32 to 133 points), with higher points indicating higher readiness was calculated. Likert-scales were re-coded to create two categories of readiness (lower and higher). For the 4-point Likert-scale items, scores 1 and 2 were re-coded as lower readiness, and scores 3 and 4 were re-coded as higher readiness. For subscale 5, scores 1 and 2 were coded as lower readiness, score 3 as “unchanged”, and scores 4 and 5 as higher readiness. Respondents answering the middle alternative (unchanged) were excluded in the total subscale 5 assessment of higher/lower readiness. Scoring and coding were carried out in accordance with a previous study using E-Ready 2.0 [25].

Groups were created by labor ward (1–10), sex (male, female), profession (assistant nurse, midwife, physician, other), employment rate (1%-49%, 50%-74%, 75%-99%, 100%), time at current workplace (0–2 years, 2–5 years, 5–10 years, 10 or more years), time in current profession (0–2 years, 2–5 years, 5–10 years, 10 or more years), labor ward size (<2000 births annually, >2000 births annually) and type of hospital (university, non-university).

### Data collection on implementation fidelity

Data on fidelity were assessed by the degree of documentation within the LCG-SE tool. A coding protocol was generated for the aims of this study. The protocol covered all seven sections and 32 parameters within the LCG-SE tool.

First, all parameters in the LCG-SE tool were assessed and discussed within the research group (JS, ARI, MB) to determine the most appropriate assessment approach, based on the guidelines in the Swedish user’s manual and clinical relevance. Parameters were categorized as: (1) having *clear recommendations* on documentation frequency; (2) having *no recommendations* on frequency, but should be documented at least once; and (3) are *sometimes not to be filled*; i.e., risk factors or medication (see Additional file 1 for details). Parameters with clear recommendations (group 1) were assessed as degree of expected documentation (0%-100%), parameters to be documented at least once (group 2) were assessed dichotomously (yes = 100% or no = 0%), and the parameters that were not always applicable (group 3) were also assessed dichotomously but excluded when calculating fidelity per section and total fidelity score.

To confirm reliability in the measure, the coding protocol was pilot tested by having two researchers (JS, ARI) assessing about five LCG-SE cases separately followed by discussions on how to interpret the documentation degrees. No modifications were made to the coding protocol. One researcher (JS), then carried out coding for all LCG-SE cases. Fidelity was assessed at 3, 6, and 9 months after implementation launched. For each time point (+4 weeks), 25 LCG-SE cases were randomly selected at each of the ten participating labor wards, resulting in 250 cases at each time point and 750 cases in total. Exclusion criterion was cases with a total documentation time shorter than four hours, since this time interval was considered necessary based on clinical relevance to ensure a necessity of documenting all parameters.

A total LCG-SE fidelity score of all 24 parameters (groups 1 and 2) was analyzed by summing the value of each parameter and calculating a mean degree value (range 0 to 100) with higher score indicating higher fidelity. Alike, sum scores were calculated for parameters considered core components (sections Supportive Care and Shared Decision-Making) and for each of the seven sections. Groups were created as for organizational readiness by labor ward, labor ward size, and hospital type, and by time point (3, 6, and 9 months).

To assess whether organizational readiness was associated with fidelity, the aggregated mean at ward level on E-Ready 2.0, and the degree of fidelity to core components at each site were used. Core components were chosen as the primary outcome, since local contextual differences could have influenced how other parts of the tool were used, with consequences for the total fidelity score. Group comparisons were deemed as most appropriate statistical approach, since measures were collected at ward level rather than individual level. Three groups were created based on the aggregated mean E-Ready 2.0 score: the three wards with the highest scores, the four wards with intermediate scores, and the three wards with the lowest scores. In addition, as a sensitivity analysis, the association was also analyzed by assessing the E-Ready 2.0 scores as a continuous variable.

### Statistical analysis

Statistical analysis was performed by the Statistical Package for the Social Science (SPSS), version 30 for Windows. Normality of data was assessed via histograms and interpreted normally distributed for data on E-ready 2.0 and total fidelity. Thus data on fidelity to core components were not normally distributed.

Descriptive statistics, with percentages or frequencies, were used to analyze background information and data on E-Ready 2.0 and fidelity scores.

Since all data were not normally distributed, Levene’s test for homogeneity was run between groups to decide the best suitable test for each group comparison resulting in t-test or Mann-Whitney U test if two groups, and ANOVA or Kruskal-Wallis test if more than two groups. For parametric tests, post hoc analyses with Tukey´s were run, and for non-parametric tests, Bonferroni corrections were used. For the sensitivity analysis, a linear regression analysis was run between the individual E-Ready scores and fidelity to the core components. Significance level was set at 0.05.

## Results

This study included ten labor wards and comprised 282 questionnaires (278 completed questionnaires) out of 904 possible, response rate 31.2%, as well as 750 reviewed LCG-SE cases. The mean duration of the documentation in the LCG-SE cases, from onset to birth, was 7.6 h (range 4–25 h). Further characteristics are presented in Tables 1 and 2.

**Table 1:** Characteristics of labor wards and mean documentation time in LCG-SE cases

Ward	Annual births (*n*)*	Log-in access/ logged in (*n*)**	Staff attending education (*n*)	Hospital type	E-Ready 2.0 *n* (rr %)	LCG-SE duration h (min–max)	First stage duration h (min–max)	Second stage duration h (min–max)
1	1895	172/ 96	50	County	20 (20.8)	8.4 (4–18)	6.5 (1–15)	1.9 (0–5)
2	1382	136/ 82	94	County	27 (32.9)	9.1 (4–20)	6.6 (1–19)	2.5 (0–9)
3	3719	213/ 151	167	Regional	33 (21.9)	8.0 (4–25)	5.8 (1–23)	2.2 (0–6)
4	2054	239/ 153	118	Regional	17 (11.1)	6.8 (4–20)	5.0 (1–16)	1.8 (0–5)
5	1106	118/ 68	61	County	38 (55.9)	8.0 (4–18)	5.6 (1–15)	2.4 (0–11)
6	3062	209/ 87	116	University	39 (44.8)	7.1 (4–17)	4.5 (1–17)	2.2 (0–7)
7	1683	171/ 92	79	University	38 (41.3)	7.8 (4–18)	5.7 (1–16)	2.1 (0–5)
8	736	91/ 51	28	County	22 (43.1)	7.3 (4–18)	5.5 (1–17)	1.8 (0–6)
9	817	84/ 64	65	County	29 (45.3)	7.4 (4–20)	4.8 (1–19)	2.6 (0–8)
10	1688	91/ 60	68	Regional	19 (31.7)	6.1 (4–13)	4.7 (1–11)	1.4 (0–4)
TOTAL	18,132	1524/ 904	846		282 (31.2)	7.6 (4–25)	5.5 (1–23)	2.1 (0–11)

*Statistics from 2023, the Swedish pregnancy register, **Number of staff on site who have log-ins to the LCG-SE platform/ Number of staff that had logged in to the LCG-SE platform at least once during data collection period, n = number, h = hours, rr = response rate

**Table 2:** Characteristics of 282 respondents to E-Ready 2.0

Respondent characteristics E-Ready 2.0	
**Sex**	**n (%)**
Male	12 (4.3)
Female	269 (95.4)
Do not want to answer	1 (0.4)
**Age**	**n years**
Mean ± SD	44.6 ± 10.6
Min-Max	20-75
**Profession**	**n (%)**
Assistant nurse	19 (6.7)
Midwife	175 (62.1)
Physician	82 (29.1)
Other	6 (2.1)
**Employment rate**	**n (%)**
1%–49%	11 (3.9)
50%–74%	16 (5.7)
75%–99%	78 (27.7)
100%	177 (62.8)
**Years at current workplace**	**n (%)**
0–2	69 (24.5)
2–5	61 (21.6)
5–10	78 (27.7)
10 or more	74 (26.2)
Mean ± SD	8.2 ± 7.3
Min-Max	0-36
**Years in current profession**	**n (%)**
0–2	38 (13.5)
2–5	56 (19.9)
5–10	49 (17.4)
10 or more	139 (49.3)
Mean ± SD	12.8 ± 9.9
Min-Max	0-43

n = number

### Organizational readiness

The overall mean E-Ready 2.0 score for all respondents was 98.5, with 75.4% reporting high readiness. The subscales with the highest proportion of respondents reporting high readiness were workplace attitudes (86.7%) and conditions for change at the workplace (82.6%). In contrast, the lowest proportions were observed for support from management (61.3%) and consequences on status quo (68.6%). This indicates highest readiness reported regarding Motivation and Capacity for change, whereas lowest readiness regarding Innovation characteristics and Leadership support, see Table 3.

**Table 3:** Organizational readiness by subscale, item and total score in E-Ready 2.0 questionnaire

Subscale in E-Ready 2.0 (*n*)	Item in E-Ready 2.0	Lower readiness *n* (%)	Higher readiness *n* (%)	Readiness per subscale Lower/Higher %
1. Conditions for change at the workplace (282)	Sufficient allocated time to implement LCG-SE	71 (25.2)	211 (74.8)	17.4/ 82.6
	Sufficient staff resources to implement LCG-SE	74 (26.2)	208 (73.8)	
	Clear goals how to implement	39 (13.8)	243 (86.2)	
	Experience among colleges working with digital solutions	39 (13.8)	243 (86.2)	
	Experience among colleges to implement new work routines	35 (12.4)	247 (87.6)	
	Adequate competency to implement LCG-SE	21 (7.4)	261 (92.6)	
	Sufficient support and guidance to adapt LCG-SE to our workplace	62 (22.0)	220 (78.0)	
	Sufficient training to be able to use and work according to LCG-SE	51 (18.1)	231 (81.9)	
2. Individual conditions for change (281)	Experience to work with digital solutions	18 (6.4)	263 (93.6)	21.5/ 78.5
	Competency to work with LCG-SE	71 (25.3)	210 (74.7)	
	Feel comfortable working with LCG-SE	92 (32.7)	189 (67.3)	
3. Support and engagement among management (280)	Communicates the need for implementing LCG-SE	123 (43.8)	158 (56.2)	38.7/ 61.3
	Encourages staff to engage in activities to implement LCG-SE	81 (28.8)	200 (71.2)	
	Takes an active role in implementing LCG-SE	115 (40.9)	166 (59.1)	
	Clearly communicates with staff how LCG-SE will be implemented	116 (41.3)	165 (58.7)	
	Have insights in how LCG-SE will influence status quo	108 (38.6)	172 (61.4)	
4. Readiness among colleagues (279)	Discuss how work routines need to change when implementing LCG-SE	58 (20.8)	221 (79.2)	27.2/ 72.8
	Discuss about duties that need to be omitted when implementing LCG-SE	135 (48.4)	144 (51.6)	
	Discuss about new duties that needs to be done when implementing LCG-SE	82 (29.4)	197 (70.6)	
	Work together to adapt current work routines to LCG-SE	59 (21.1)	220 (78.9)	
	Take a collective responsibility for the implementation	45 (16.1)	234 (83.9)	
5. Consequences on status quo (278)	Your ability to work in accordance with your values	55 (19.8)	139 (50.0)	31.4/ 68.6
	Your ability to exercise your professional role	24 (8.6)	123 (44.2)	
	Your ability to offer high quality care	19 (6.8)	142 (51.1)	
	Your ability to manage all your work	83 (29.9)	48 (17.3)	
	Your current routines at your workplace	72 (25.9)	123 (44.2)	
6. Workplace attitudes (278)	Among colleagues	54 (19.4)	224 (80.6)	13.3/ 86.7
	Your attitude	39 (14.0)	239 (86.0)	
	Your manager’s attitude	18 (6.5)	260 (93.5)	
7. Compatibility with current work routines (278)	How well does LCG-SE fit with current work routines?	62 (22.3)	216 (77.7)	
8. Commitment to change (278)	What role would you like to have when LCG-SE is being implemented at your workplace?	82 (29.5)	196 (70.5)	
9. Perceived need for change (278)	How do you perceive the value of implementing LCG-SE at your workplace?	56 (20.1)	222 (79.7)	
**Total readiness score (278)**	Total score, all items in E-Ready 2.0			24.6/ 75.4 98.5 ± 15.4*

*Total score for all items in E-Ready 2.0, possible range from 32 to 133 points (higher score indicating higher readiness) ± SD, n = number

### Fidelity to documentation in LCG-SE

The degree of documentation for specific parameters differed markedly. Parameters assessed by degree of expected documentation ranged from a mean of 25.4% (parameter 23., Duration of contractions in second stage of labor) to 91.5% (parameter 25., Station); and for parameters assessed dichotomously from 27.2% (parameter 12., Pulse) to 100.0% (parameter 26., Cervix). The mean overall documentation rate across all 24 parameters included in the total LCG-SE score was 70.7%, while the 7 core component parameters showed a higher mean of 83.4%. The sections with the highest documentation degree were Background and labor characteristics (Section  1), Supportive Care (Section  2, core component) and Shared Decision-Making (Section  7, core component), while the section with lowest degree was Labor Progress (Section  5), see Table 4.

**Table 4:** Fidelity to documentation in LCG-SE, by parameter, total score LCG-SE and core components

Section in LCG-SE	Parameter in LCG-SE	Assessed dichotomously, documented at least once Yes/No n (%), *n* = 750	Assessed by degree of expected documentation % ± SD, *n* = 750	Degree of documentation per section % ± SD
1. Background and labor characteristics	1. Parity	746/4 (99.5/0.5)		98.7 ± 5.6
	2. Week of pregnancy	749/1 (99.9/ 0.1)		
	3. Labor onset	745/5 (99.3/0.7)		
	4. Ruptured membranes	722/28 (96.3/3.7)		
	5. Risk factors*	594/156 (79.2/20.8)		
2. Supportive Care**	6. Presence Companion		91.4 ± 14.6	87.2 ± 15.9
	7. Presence healthcare personnel		87.3 **±**18.0	
	8. Pain relief*	747/3 (99.6/0.4)		
	9. Pain situation		87.9 ± 16.6	
	10. Oral fluids		82.7 ± 21.6	
	11. Posture		86.7 ± 18.8	
3. Woman	12. Pulse	204/546 (27.2/72.8)		56.3 ± 26.0
	13. Blood pressure	375/375 (50.0/50.0)		
	14. Temperature °C	389/361 (51.9/48.1)		
	15. Urine	720/30 (96.0/4.0)		
4. Baby	16. Fetal Heart Rate 17. CTG	743/7 (99.1/0.9)		70.7 ± 22.0
	18. Amniotic fluid		71.7 ± 34.0	
	19. Caput		41.2 ± 41.8	
5. Labor Progress	20. Contractions, first stage		40.5 ± 26. 9	50.0 ± 14.0
	21. Contractions, sec stage		34.6 ± 31.3	
	22. Dur contractions, first stage		28.0 ± 26.7	
	23. Dur contractions, sec stage		25.4 ± 31.8	
	24. External palpation		30.3 ± 33.2	
	25. Station		91.5 ± 18.7	
	26. Cervix	750/0 (100.0/0.0)		
	27. Fully dilated*	691/59 (92.1/7.9)		
	28. Pushing*	467/283 (62.3/37.7)		
6. Medication	29. Oxytocin*	513/237 (68.4/31.6)		
	30. Medicine*	581/169 (77.5/22.5)		
7. Shared Decision-Making**	31. Assessment		71.1 ± 33.7	75.2 ± 24.4
	32. Plan		76.9 ± 29.5	
**1 Total score LCG-SE**	Fidelity to 24 parameters in LCG-SE			70.7 ± 9.0
**2 Core Components**	Fidelity to 7 core components			83.4 ± 15.9

*Parameter in group 3 in coding protocol, excluded from fidelity assessment per section and total score, **Core component section, n = number, dur = duration

### The association between organizational readiness and implementation fidelity

The wards with the aggregated highest E-Ready 2.0 scores were wards 1, 2, and 5 (readiness score 102–109; *n* = 85). The intermediate aggregated scores included wards 3, 7, 8, and 9 (readiness score 98–99; *n* = 122), and the lowest aggregated scores included wards 4, 6, and 10 (readiness score 88–96; *n* = 75).

There were statistically significant differences between these groups in terms of degree of fidelity to the core components. The wards with the aggregated lowest readiness scores also demonstrated the lowest degree of fidelity in documenting core component parameters, while wards with the aggregated highest readiness score also had the highest degree of fidelity, see Table 5.

**Table 5:** The association between organizational readiness and implementation fidelity

Group	Fidelity to Core Components Median (IQR)	Difference between groups	Post-hoc analysis
1. Lowest aggregated readiness score	81.6 (76.5–81.6)	***p*** < 0.001*	1 vs. 2, *p* = 0.00**
2. Intermediate aggregated readiness score	83.5 (83.5–86.4)		2 vs. 3, *p* = 0.02**
3. Highest aggregated readiness score	85.1 (81.1–90.3)		1 vs. 3, *p* = 0.00**

*Independent samples Kruskal-Wallis test, **Pairwise comparison adjusted by Bonferroni correction

Additionally, the sensitivity analysis by linear regression analysis showed significant differences at individual level with unstandardized mean difference B = 0.65 (95% CI = 0.13–1.17, p-value = 0.02). Thereby the association between organizational readiness and implementation fidelity remained significant and in the same direction, supporting the robustness of the primary findings.

### Variations across organizational and individual factors and over time

Group differences based on readiness (total scores) were observed where staff working at larger wards (>2000 births annually) reported lower readiness than staff working at smaller wards (<2000 births annually). There were also differences based on hospital type, where university hospital staff reported lower readiness than staff at non-university hospitals. Between professions, midwives reported lower readiness than physicians, and for time in current profession, the group representing 5–10 years reported lower readiness than those who had worked for 2–5 years in their current profession, see Table 6.

**Table 6:** Differences between groups in perceptions of organizational readiness based on E-Ready 2.0

Group (n)	Total score E-Ready 2.0 Mean ± SD	95% CI	Difference between groups	Post Hoc analysis	
**Labor ward**				Groups	*p*-value
1 (19)	109.2 ± 8.3	105.2–113.2	***p*** < 0.001**	1 vs. 6	**0.00**
2 (27)	102.3 ± 18.3	95.1–109.6		2 vs. 6	**0.00**
3 (33)	99.1 ± 16.5	93.3–105.0			
4 (17)	95.7 ± 19.2	85.8–105.6			
5 (37)	103.5 ± 13.9	98.9–108.1		5 vs. 6	**0.00**
6 (38)	88.0 ± 12.2	84.0–92.1			
7 (38)	98.2 ± 15.2	93.2–103.2			
8 (22)	99.7 ± 14.1	93.5–106.0			
9 (29)	98.3 ± 13.0	93.4–103.2			
10 (18)	93.8 ± 13.7	87.0–100.6			
**Sex**					
Male (12)	98.6 ± 15.5	90.6–106.1	*p* = 0.96*		
Female (265)	98.3 ± 13.3	96.7–100.3			
**Profession**				Group	*p*-value
1. Nursing assistant (18)	96.1 ± 19.0	86.7–105.5	***p *** **=0.03¤**		
2. Midwife (174)	96.7 ± 15.2	94.4–99.0		2 vs. 3	**0.02**
3. Physician (81)	102.8 ± 14.3	99.7–106.0			
4. Other (5)	98.8 ± 16.7	78.1–119.5			
**Employment rate**					
1%–49% (11)	102.0 ± 16.7	90.8–113.3	*p* = 0.16¤		
50%–74% (16)	95.7 ± 16.8	86.7–104.6			
75%–99% (77)	95.5 ± 15.9	91.9–99.1			
100% (174)	99.8 ± 14.9	97.6–102.0			
**Time at workplace**					
0–2 years (68)	98.6 ± 17.6	94.4–102.9	*p* = 0.66¤		
2–5 years (60)	100.3 ± 15.3	96.3–104.3			
5–10 years (76)	96.9 ± 14.9	93.5–100.3			
≥10 years (74)	98.5 ± 14.0	95.2–101.7			
**Time in profession**				Group	*p*-value
1. 0–2 years (38)	98.3 ± 17.6	92.5–104.03	***p *** **=0.04¤**		
2. 2–5 years (55)	103.0 ± 15.0	98.9–107.0		2 vs. 3	**0.02**
3. 5–10 years (47)	94.2 ± 15.8	89.5–98.8			
4. ≥10 years (138)	98.2 ± 14.5	95.8–100.7			
**Labor ward size**					
>2000 births (88)	93.7 ± 16.0	90.2–97.2	***p*** < 0.001*		
<2000 births (190)	100.7 ± 14.6	98.7–102.8			
**Hospital type**					
University (76)	93.1 ± 14.7	89.8–96.2	***p*** < 0.001*		
Non-University (202)	100.5 ± 15.3	98.3–102.6			

*Independent samples t-test for equality of means, two-sided, **Independent samples Kruskal-Wallis test with Pairwise comparison adjusted by Bonferroni correction, ¤ANOVA for multiple comparison with Tukey HSD

Group comparisons of implementation fidelity showed differences depending on whether fidelity was assessed for the total LCG-SE score or for the core components. Labor wards with more than 2000 births annually had lower fidelity to both the LCG-SE total score and to the core components compared to wards with less than 2000 births annually. For the total LCG-SE score, there was a significant change in documentation fidelity over time, with higher fidelity at three months compared to nine months after implementation. No similar change was found for the core components, see Table 7.

**Table 7:** Differences between groups in implementation fidelity to LCG-SE documentation

Group (n)	1. LCG-SE Mean ± SD (95% CI)	2. Core Components Mean ± SD (95% CI)	Differences between groups 1. LCG-SE 2. Core components	Post Hoc analysis 1. LCG-SE		Post Hoc analysis 2. Core Components	
**Labor ward **				Group	*p*-value	Group	*p*-value
1 (75)	71.2 ± 8.6 (69.3-73.1)	85.1 ± 14.4 (81.9–88.2)	1. ***p* = 0.00**⁑ 2. ***p* < 0.001**⁑				
2 (75)	71.7 ± 6.8 (70.3-73.2)	90.3 ± 9.6 (88.1-92.5)				2 vs. 10	**0.00**
3 (75)	68.4 ± 9.4 (66.2-70.4)	83.5 ± 13.7 (80.2-86.4)		3 vs. 8	**0.01**		
4 (75)	70.2 ± 10.4 (67.8-72.6)	79.6 ± 19.6 (74.8-84.0)				2 vs. 4	**0.03**
5 (75)	70.6 ± 8.5 (68.7-72.3)	81.1 ± 16.4 (77.2-84.4)				2 vs. 5	**0.01**
6 (75)	68.0 ± 8.8 (66.1-70.1)	81.6 ± 16.3 (77.8-85.3)		6 vs. 8	**0.00**	2 vs. 6	**0.04**
7 (75)	71.9 ± 8.3 (69.9-73.7)	86.4 ± 13.8 (83.2-89.5)				7 vs. 10	**0.01**
8 (75)	73.7 ± 7.0 (72.0-75.2)	86.8 ± 13.7 (83.4-89.8)				8 vs. 10	**0.00**
9 (75)	69.7 ± 9.6 (67.4-72.0)	83.4 ± 18.1 (79.3-87.2)					
10 (75)	71.2 ± 10.5 (68.7-73.6)	76.5 ± 17.8 (72.4-80.5)					
**Labor ward size**							
>2000 births (225)	68.9 ± 9.6 (67.6-70.1)	81.6 ± 16.7 (79.4-83.7)	1. *p* < 0.001** 2. *p* = 0.05**				
< 2000 births (525)	71.4 ± 8.6 (70.7-72.2)	84.2 ± 15.6 (82.7-85.6)					
**Hospital type**							
University (150)	67.0 ± 8.8 (68.6-71.5)	84.0 ± 15.2 (81.6-86.4)	1. *p* = 0.29* 2. *p* = 0.62*				
Non-University (600)	70.8 ± 9.0 (70.1-71.6)	83.3 ± 16.1 (82.0-84.7)					
**Over time**				Group	*p*-value		
1. 3 months (250)	71.9 ± 8.7 (70.9–73.0)	83.3 ± 15.5 (81.4-85.2)	1. ***p* = 0.01¤** 2. *p* = 0.99¤	1 vs. 3	**0.01**		
2. 6 months (250)	70.6 ± 8.7 (69.6–71.7)	83.4 ± 15. 8 (81.6-85.4)					
3. 9 months (250)	69.4 ± 9.3 (68.2–70.5)	83.5 ± 16.6 (81.4-85.5)					

*Independent samples t-test for equality of means, two-sided, ⁑Independent samples Kruskal-Wallis test, with Pairwise comparison adjusted by Bonferroni correction, **Independent samples Mann-Whitney U test, ¤ANOVA for multiple comparison, with Tukey HSD, n = number

## Discussion

This study aimed to examine whether organizational readiness to implement a digital documentation tool (the LCG-SE) within Swedish labor care was associated with implementation fidelity at 3, 6, and 9 months. We also investigated variations across organizational and individual factors as well as change over time. The results showed that organizational readiness was associated with implementation fidelity in this high-income context. In addition, hospital type, labor ward size, and profession influenced the level of organizational readiness. Although fidelity for the complete documentation tool (all parameters) decreased over time, this was not observed for the core components (parameters new for this intervention), where fidelity was stable over time.

The precise role of organizational readiness in implementation is often described as unclear [11]. There is a lack of validated readiness measures as well as consensus about the components within the readiness concept. Therefore, enhanced collaboration between implementation science and practice to achieve greater clarity and theoretical relevance to the concept is needed [11]. By empirically linking organizational readiness with implementation outcomes, the present study addresses a gap in existing implementation research. Our results are comparable with earlier studies that have linked readiness to early implementation outcome measures [13–15], and our findings support the theoretical premise that organizational readiness contributes to implementation outcomes and thereby strengthens its conceptualization.

Most respondents in our study reported high levels of organizational readiness. The multi-dimensional construct of organizational readiness, encompassing both willingness and capability, however, makes it difficult to draw conclusions only based on readiness levels. Total scores are valuable within research to investigate determinants of organizational readiness or findings across studies; however a total score of organizational readiness may be less helpful when trying to understand how ready an organization, work group, or clinic is to implement a specific change. When planning implementation strategies, the different dimensions of organizational readiness need to be considered.

For illustration, E-Ready 2.0 has previously been applied in one study investigating digital health readiness among healthcare leaders in operational management in South Africa [25]. Similar to our findings, the subscale *workplace attitude* (Motivation) had the highest proportion of respondents reporting high readiness. The remaining subscales, however, differed. In our study, *conditions for change at workplace* (Capacity) had the second-highest proportion of high readiness, whereas leaders in South Africa reported this subscale among the lowest. Conversely, one of the lowest-rated subscales in our context was *consequence on status quo* (Innovation Characteristics), while leaders in South Africa reported this subscale among the highest. These findings support the notion that organizational readiness is context- and innovation specific. An organization where the willingness to change is high, but lacks the necessary capability, is just as unprepared as one that is well-equipped but lacks willingness to change among staff. Assessing different dimensions of organizational readiness (e.g., capacity for change versus willingness to change) can therefore be important when planning implementation efforts and deciding upon implementation strategies. In a high resource setting, such as Swedish maternity care, supportive leadership that motivates the characteristics of the innovation may be more critical to facilitate implementation than, for example, workplace capacity.

Furthermore, our findings suggest that even within a seemingly homogeneous context, such as Swedish maternity care, perceptions of readiness are shaped by both organizational (e.g., ward size or hospital type) and individual (e.g., profession or time in current profession) factors. These results align with some previous studies, while contradicting others. The underlying reasons for these discrepancies remain insufficiently investigated. However, a study among emergency nurses in Switzerland [26] concluded that organizational factors such as work environment and supportive leadership were associated with higher organizational readiness, which may help to explain some of the variation observed.

Regarding individual factors, no differences between professional groups were found when assessing organizational readiness for a new midwifery model of care in rural South Australia [27], whereas such differences emerged in the present study. Other studies comparing educational attainment have shown that nurses with higher educational levels or university degrees tend to report higher organizational readiness [26], and both nurses and managers with more work experience report higher organizational readiness than those who were newer to the field [25, 26]. These findings differ slightly from the present study, again reinforcing that organizational readiness is a multidimensional construct. Taken together, the evidence suggests that both psychological and structural dimensions of readiness must be considered before initiating any implementation effort.

Regarding implementation fidelity, the present study is the first to quantify fidelity to the complete LCG-SE tool and to assess its core components. Although one study in Uganda assessed completion rates for selected parts of LCG during an implementation effort [6]. The overall completion rate in that context was lower than expected, although still higher than with the previous documentation tool. In the present study, the expected documentation rate for the LCG-SE was about three quarters, and a considerable variation was observed across different parameters. Core components were generally documented to a higher degree than non-core elements.

One possible explanation is that decisions made at the local level may have resulted in certain parameters being documented elsewhere. Another explanation is that some parameters may have already been inconsistently documented in previous Swedish documentation practices. Since no direct comparison was conducted, these potential explanations remain speculative. Nevertheless, by separately assessing the core components, the present study provides a more nuanced understanding of what is actually implemented and sustained in practice. These findings underscore the importance of identifying and anticipating the role of core components when preparing an innovation. Ensuring that such components are fully adopted and adequately implemented is crucial; otherwise, evaluations risk committing a so-called Type III error, (assessing clinical outcomes of an innovation that was never truly implemented) [20].

Another noteworthy finding concerns fidelity over time. The longitudinal assessment of fidelity, conducted at three time points, provides early indications of how implementation dynamics evolve. Although overall LCG-SE fidelity decreased over time, fidelity to the core components remained stable. This is a central insight, even if the mechanisms behind it remain unclear. Higher fidelity to core components is expected [13], which may partly explain this pattern.

Despite this, international guidelines from FIGO and WHO emphasize that the LCG should be used as a complete tool, with every parameter consistently and accurately recorded to prevent complications [2, 3]. Furthermore, it has been suggested that improving completion rates could improve labor and delivery outcomes [6]. Given that the present study indicates that some sections and parameters are used more frequently than others, future efforts should focus on designing a version of the LCG that ensures greater usability and sustainability, that promotes complete documentation. Ideally, such improvements could contribute to better implementation outcomes, intrapartum care and outcomes for both women and their babies.

### Strengths and limitations

This study contributes to the field of implementation science in several important ways. By using appropriate and rigorous methods to address the research questions and to evaluate the implementation process, its findings give empirical support to the premise that organizational readiness is associated with concrete implementation outcomes. In doing so, it responds directly to repeated calls within implementation science for a stronger link between theory and clinical practice. The study therefore offers both practical and theoretical value to the field.

Furthermore, the inclusion of multiple labor wards, from varying types of hospitals and ward sizes as well as different professions, implementing the same innovation simultaneously strengthens internal validity and allows meaningful comparisons across real-world organizational settings. The design increased heterogeneity, thereby strengthening the relevance of the findings for maternity care implementation in high resource settings. This diversity further enables comparisons with international studies using the same instrument and illustrates how contextual factors shape readiness. Taken together, the findings reinforce that organizational readiness is both multidimensional and context-dependent, adding depth to the interpretation.

However, the study also has limitations. First, the observational, cross-sectional design limits the ability to control for external factors. Therefore, identifying a statistical relationship between variables is insufficient to conclude causality.

Furthermore, both the sample representativeness and sample size for the questionnaire warrant careful consideration. Neither sample size nor statistical power were estimated in advance. The total response rate was low, leading to a risk of non-response bias. However, the fact that the response rate showed a wide dispersion between the wards, varying from about 11% to 56%, and no pattern was observed suggesting that higher or lower response rates were associated with higher or lower readiness scores, do indicate that this concern had small impact on the results of this study. Further, no non-response analysis was made, making it impossible to determine the true response rate. Non-respondents may have been employees who were off duty during data collection, or individuals who were not eligible to take part.

Although the E-Ready 2.0 has previously been tested and considered sensitive for use in Swedish contexts, the design of the PICRINO trial, with a short introduction time for the intervention, created challenges in identifying the optimal time point for measuring organizational readiness in accordance with Weiner’s organizational readiness theory [9]. This introduces a further risk of response bias. Further, given that organizational readiness is a dynamic and evolving construct, assessing it only once offers limited insight into temporal variations or readiness trajectories. Multiple measurement points could have mitigated some of these concerns and allowed the detection of changes over time.

Regarding the interpretation of fidelity, additional limitations emerge from the study design. First, this study defines fidelity as the degree of documentation, rather than the accuracy of the documentation. There is a possibility that the health care staff did document frequently, but with low quality and therefore the assumption that a high documentation rate implies high fidelity might be slightly strong. Further, the results primarily address the *exposure* component in the fidelity framework [21], while paying less attention to the other three components. This focus is, however, common in clinical research where studies typically emphasize the quantity, rather than the quality, of fidelity [28]. A potential strength would have been to assess the component *participant responsiveness* in the fidelity framework further or even to include additional outcome measures, such as acceptability, appropriateness, feasibility, or sustainability, to enable comparison across multiple types of outcomes in relation to organizational readiness.

### Implications for future research and perspectives

This study contributes both practical and theoretical value to the field of implementation science. Nevertheless, there are still some questions left unanswered and important implications for future research should be highlighted. For example, although this study demonstrates an association between organizational readiness and fidelity, it does not clarify *which levels* of readiness (e.g., individual, team, unit, or organizational) matters most, nor *how ready* an organization should be before initiating a change. Nor does this study answer whether all members of an organization need to be ready at the same time, since this study only had one measurement point. To advance the field, future implementation research should therefore measure organizational readiness prospectively, across multiple stages of implementation, and examine the thresholds at which readiness yields optimal effects on implementation outcomes.

Our results do indicate that fidelity decreases over time. Due to the study design of the PICRINO trial long-term implementation outcomes, such as sustainability, could not be evaluated. Therefore, in the event of a nationwide rollout of the LCG-SE in Sweden or comparable high resource settings, implementation research should focus especially on long-term implementation outcomes. It is essential to understand whether the patterns observed in this study persist beyond nine months or whether the initial decline stabilizes or even reverses over time. Such knowledge would be essential for guiding the design of more durable implementation strategies for the LCG-SE and similar innovations.

## Conclusions

This study provides empirical support for an association between organizational readiness and implementation fidelity when introducing the digital Labour Care Guide in Swedish labor wards. Furthermore, the findings indicated that both organizational and individual factors influence organizational readiness. Fidelity to the Labour Care Guide decreased over time; however, fidelity to core components remained stable, suggesting that essential elements of the intervention were more resilient to implementation challenges.

In the context of a potential nationwide rollout of the tool, or in comparable settings involving similar adaptations of the Labour Care Guide, the findings highlight the importance of assessing and strengthening organizational readiness prior to implementation and of providing sustained support over time to maintain fidelity, particularly for non-core components. Strategies tailored to organizational context and professional roles may be necessary to optimize long-term implementation success of complex digital clinical tools. Such preparedness is essential for optimizing implementation and, ultimately, for contributing to improved health care outcomes within maternity care.

## Supplementary Information

Below is the link to the electronic supplementary material.

Supplementary Material 1

## Data Availability

The datasets used and/or analyzed during the current study are available from the corresponding author upon reasonable request.

## Abbreviations

FIGO: The International Federation of Gynecology and Obstetrics
LCG: Labour Care Guide
LCG–SE: Labour Care Guide–Sweden
WHO: World Health Organization
