# Supplementary material for: Associations between organizational readiness and implementation fidelity in national implementation of WHO’s labour care guide in Sweden: a prospective observational study
Source: Implement Sci Commun. 2026 Aug 14;7:165. doi: 10.1186/s43058-026-01073-z (PMC13501707; doi:10.1186/s43058-026-01073-z)
Supplement: Supplementary file 1 [file 43058_2026_1073_MOESM1_ESM.docx]

| Coding protocol for implementation fidelity to documentation in LCG-SE | |
| --- | --- |
| General instructions:  Assessment of each parameter is conducted from the initiation of LCG-SE until its completion. Parameters are categorized as; Group 1* having recommendations specified in the Swedish user’s manual on documentation frequency or is based on clinical relevance, Group 2** having no recommendations on frequency in the Swedish user’s manual, but should be documented at least once, and Group 3*** are patient-specific and sometimes not to be filled.  **Assessment grounds:**  **Inclusion criteria**: LCG-SE case ongoing for a minimum of four full hours, including active first stage and active second stage of labor.  **If the first and/or last hour is “partial”** (i.e., LCG-SE case was not initiated or finished on the hour), these hours are excluded for variables that are assessed on a time basis (hourly, half-hourly, or quarterly). This exclusion does not apply on dichotomously assessed parameters.  **If the transition from the active first stage to the active second stage** does not occur on the hour, the final hour of the first active stage is merged with the first hour of the active second stage for parameters assessed on hourly basis. This does not apply on dichotomously assessed parameters. | |
| **Parameter** | **Instruction and assessment** |
| **Section 1 Background**  General instruction: Each parameter should be documented once. | |
| 1. Parity** | Parity noted Yes/No |
| 2. Week of pregnancy** | Week of pregnancy noted Yes/No |
| 3. Labor onset ** | Labor onset noted Yes/No |
| 4. Ruptured membranes** | Ruptured membranes noted in section 1 or 4 Yes/No |
| 5. Risk factors*** | Risk factors noted Yes/No |
| **Section 2 Supportive Care**  General instruction: Each parameter (except for item 8) should be documented once hourly. Core component. | |
| 6. Presence companion* | Number of companion notes divided by number of LCG-SE hours |
| 7. Presence healthcare personnel* | Number of healthcare personnel notes divided by number of LCG-SE hours |
| 8. Pain relief*** | Pain relief noted at least once Yes/No |
| 9. Pain situation* | Number of pain situation notes divided by number of LCG-SE hours |
| 10. Oral fluids* | Number of oral fluids notes divided by number of LCG-SE hours |
| 11. Posture* | Number of posture notes divided by number of LCG-SE hours |
| **Section 3 Woman**  **General instruction:** Each parameter should be documented at least once. | |
| 12. Pulse** | Pulse noted at least once Yes/No |
| 13. Blood pressure** | Blood pressure noted at least once: Yes/No |
| 14. Temperature °C** | Temperature noted at least once; Yes/No |
| 15. Urine** | Urine noted at least once; Yes/No |
| **Section 4 Baby**  **General instruction:** Includes both group 1 and 2 parameters, instructions under each parameter. | |
| 16. Fetal Heart Rate** | Parameters 16 and 17 are combined as one assessment based on clinical relevance  Fetal Heart Rate OR CTG noted at least once Yes/No |
| 17. CTG** |  |
| 18. Amniotic fluid* | If rupture of membranes is noted, amniotic fluid should subsequently be documented at each vaginal examination.  Number of amniotic fluids notes after rupture of membranes divided by number of vaginal examinations after rupture of membranes during the active first stage. If rupture of membranes is not noted insert 99/99. |
| 19. Caput* | If rupture of membranes is noted, caput should subsequently be documented at each vaginal examination.  Number of caput notes after rupture of membranes divided by number of vaginal examinations after rupture of membranes during the active first stage. If rupture of membranes is not noted insert 99/99. |
| **Section 5 Labor progress**  General instruction: Includes both group 1, 2 and 3 parameters, instructions under each parameter. | |
| 20. Contractions per 10 minutes active first stage* | Contractions per 10 minutes is separated into active first and second stages. Should be documented at least every 30 minutes during the first stage and every 15 minutes during the second stage.  20. Number of notes of contractions per 10 minutes in the active first stage divided by number of half-hour intervals during active first stage  21. Number of notes of contractions per 10 minutes in the active second stage divided by number of quarter-hour intervals during active second stage. |
| 21. Contractions per 10 minutes active second stage* |  |
| 22. Duration of contractions active first stage* | Duration of contractions is separated into active first and second stages. Should be documented at least every 30 minutes during the first stage and every 15 minutes during the active second stage.  22. Number of notes of duration of contractions in the active first stage divided by number of half-hour intervals during active first stage  23. Number of notes of duration of contractions in the active second stage divided by number of quarter-hour intervals during active second stage |
| 23. Duration of contractions active second stage* |  |
| 24. External palpation* | External palpation should be documented at least before each vaginal examination  Number of notes of external palpation divided by number of vaginal examinations in the active first stage. |
| 25. Station* | Same as above, 24 External palpation.  Number of notes of station divided by number of vaginal examinations in the active first stage. |
| 26. Cervix** | Cervix noted at least 1 time Yes/No |
| 27. Fully dilated*** | In group 3 based on technical limitations in the digital LCG-SE  Fully dilated noted Yes/No |
| 28. Pushing*** | In group 3 based on technical limitations in the digital LCG-SE  Pushing noted Yes/No |
| **Section 6 Medication**  General instruction: The use of this section may vary depending on local guidelines. Group 3 parameters. | |
| 29. Oxytocin*** | Oxytocin noted at least once Yes/No |
| 30. Medicine*** | Medicine noted at least once Yes/No |
| **Section 7 Shared Decision-Making**  General instruction: Based on clinical relevance, this section should be documented at least once hourly. Core component. | |
| 31. Assessment* | The parameters is assessed first with number of actual documentations per parameter per number of hours. Then by compiling the parameters and assessing the number of hours with at least one registration.  31. Number of notes Assessment divided by number of LCG-SE hours  32. Number of notes Plan divided by number of LCG-SE hours  AND  Number of hours with at least one note Assessment OR Plan divided by number of LCG-SE hours |
| 32. Plan* |  |
| **Assessment of implementation fidelity:**  Implementation fidelity is a calculated percentage based on the proportion of completed parameters out of the total possible parameters. Group 1 (marked*) is assessed by percentage completion rate out of expected registrations. Group 2 (marked**) and Group 3 (marked***) are evaluated dichotomously as either documented (YES =100%) or not (NO =0%). For parameters in group 1 where the assessment may be calculated above 1.0 (100%), assessment above 1.0 is corrected to 1.0 (100%).  A total score of fidelity is calculated by the mean completion rates of all parameters in group 1 and group 2 (totally 24 parameters). A score of fidelity to the core components is calculated by mean completion rates of the parameters in group 1 in section 2 and 7 (totally 7 parameters). Fidelity to each section is calculated by the mean completion rates of all parameters in group 1 and group 2 in that section. Group 3 parameters are not included in the total score or in the section-specific calculations. | |
